# Network pharmacology and molecular docking technology for exploring the effect and mechanism of high-dose vitamin c on ferroptosis of tumor cells: A review

**DOI:** 10.1097/MD.0000000000038189

**Published:** 2024-05-17

**Authors:** Jinxiu Qu, Shuai Lu, Bing Wang, Shiwan Wang, Zhenpeng Yang, Huazhen Tang, Jia He, Yuan Zhao, Xin Wang, Xiaozhu Liu, Benqiang Rao

**Affiliations:** a Department of Gastrointestinal Surgery, Beijing Shijitan Hospital, Capital Medical University, Beijing, China; b Center of Metabolism and Nutrition of Cancer, Beijing Shijitan Hospital, Capital Medical University, Beijing, China; c Key Laboratory of Cancer FSMP for State Market Regulation, Beijing, China; d Department of General Surgery, Qilu Hospital, Shandong University, Jinan, China; e Department of Critical Care Medicine, Beijing Shijitan Hospital, Capital Medical University, Beijing, China.

**Keywords:** ferroptosis, high-dose vitamin C, metabolism, network pharmacology, tumor

## Abstract

To investigate the mechanism by which high-dose vitamin C (HVC) promotes ferroptosis in tumor cells via network pharmacology, vitamin C-related and ferroptosis-related targets were obtained from the PharmMapper and GeneCards databases, respectively, and their common targets were compared using the Venn diagram. Common targets were imported into the STRING database for protein-protein interaction analysis, and core targets were defined. Core targets were enriched for Gene Ontology terms and Kyoto Encyclopedia of Genes and Genomes pathways using the R language packages. A map of the core target-based interaction network and a map of the mechanism by which HVC regulates ferroptosis were constructed. A total of 238 vitamin C-related and 721 ferroptosis-related targets were identified, of which 21 targets were common to both. Furthermore, ALDOA, AHCY, LDHB, HSPA8, LGALS3, and GSTP1 were identified as core targets. GO enrichment analysis suggested that the main biological processes included the extrinsic apoptotic signaling pathway and pyruvate metabolic process. KEGG enrichment analysis suggested that HVC regulates ferroptosis mainly through the amino acid and carbohydrate metabolic pathways. The targets were validated by molecular docking. In conclusion, HVC may promote ferroptosis in tumor cells by regulating metabolic pathways, and there is a synergistic effect between HVC and type I ferroptosis inducers. Glycolysis-dependent tumors may be beneficial for HVC therapy. Our study provides a reference for further clinical studies on HVC antitumor therapy.

## 1. Introduction

Vitamin C (Vc), also known as ascorbic acid, is a vital water-soluble vitamin with diverse physiological roles in the human body and is commonly utilized in disease treatment. High-dose vitamin C (HVC) is recognized as a safe and cost-effective therapy. HVC antitumor therapy has been studied for nearly 50 years since it was first clinically tested as an antitumor agent by Pauling and Cameron in 1976.^[1–3]^ The utilization of HVC as a cancer treatment modality has been controversial. Much of this controversy stems from conflicting results in early clinical trials and a lack of a clear understanding of the mechanism of action of Vc. In recent years, new insights into the pharmacokinetics of Vc have provided a partial explanation for these conflicting results: high doses of Vc, rather than low doses, can induce tumor cytotoxicity. The resurgence of interest in this treatment approach has led to a rise in related clinical investigations annually.^[4]^ In view of this, it is necessary to further investigate the mechanisms of Vc antitumor therapy, explore multiple targets and mechanisms by which Vc exerts its antitumor effects, identify biomarkers for patient stratification, and develop effective combination strategies for clinical studies.

Existing studies have labeled that the mechanism of Vc-specific tumor cell killing involves pro-oxidant activity, cofactor activity, collagen synthesis, epithelial-mesenchymal transition and invasion, oxygen sensing, epigenetic regulation, and immune modulation^[4]^: HVC act as a pro-oxidant, targeting the cytotoxic effect of the redox imbalance in tumor cells. Vc increases the level of oxidative stress, leading to DNA, protein, and lipid damage in tumor cells, whereas it has less effect on normal cells. Vc acts as an iron reducer and is involved in the catalytic activity of a number of important enzymes, such as collagen synthesis, mitochondrial respiratory chain, and oxidative stress regulation. Vc affects the metabolism and survival of tumor cells by regulating the activity of these enzymes. Vc inhibits tumor infiltration and metastasis by stabilizing the collagen fiber structure. It also reverses epithelial-mesenchymal transition and reduces the migration and invasive ability of tumor cells. (d) Vc regulates the activity of the oxygen-sensing factor hypoxia inducible factor-1 (HIF-1) and inhibits tumor growth. It can degrade HIF-1 and reduce the malignant potential of tumor cells. Tumor cells are often in a hypoxic state, which makes them more sensitive to Vc. Vc induces DNA demethylation and restores the expression of tumor suppressor genes by regulating DNA demethylase activity. It also enhances histone demethylase activity and regulates the epigenetic state of cells. Vc affects several aspects of the immune response, including differentiation, activation, and enhanced cytotoxicity of immune cells. It enhances natural killer cell-mediated tumor killing, regulates T and B cell development and function, and synergizes with immune checkpoint inhibitors. These mechanisms interact with each other and work together to exert the multi-targeting effects of Vc in antitumor therapy, thereby providing potential strategies and opportunities for tumor treatment. However, these mechanisms do not fully explain the antitumor effects of Vc. However, the relevant mechanisms need to be investigated further.

Ferroptosis, a term proposed by Dixon et al in 2012, is an iron-dependent form of regulated cell death triggered by the toxic accumulation of lipid peroxides on plasma membranes.^[5]^ This process can be initiated through various pathways that modulate intracellular iron levels, amino acid metabolism, and polyunsaturated fatty acid metabolism. Ferroptosis inducers are compounds or therapeutics that can induce cellular ferroptosis, and can be categorized into four groups based on their mechanisms of action. Given the heightened intracellular iron content in tumor cells and their increased susceptibility to ferroptosis, ferroptosis inducers hold significant promise in cancer therapy. Ferroptosis plays a huge role in tumor suppression and immunity and may offer new therapeutic strategies for tumors that are difficult to treat with conventional therapies. Multiple cancer-related signaling pathways are involved in the control of ferroptosis in cancer cells.^[6]^ HVC-induced cell death shares several features with ferroptosis, including iron dependence, reactive oxygen species (ROS) production, and lipid peroxidation. Additionally, studies have shown that Vc can promote ferroptosis in mesenchymal thyroid cancer cells and sensitize pancreatic cancer cells to ferroptosis.^[7,8]^ While there is substantial evidence connecting HVC antitumor therapy with ferroptosis, the mechanisms through which HVC regulates ferroptosis remain understudied. In this study, the mechanism of ferroptosis regulation by HVC was investigated using a big data mining approach based on network pharmacology and molecular docking and discussed in the context of tumor therapy, which provides a basis for further research on HVC antitumor therapy.

**Figure 1.**
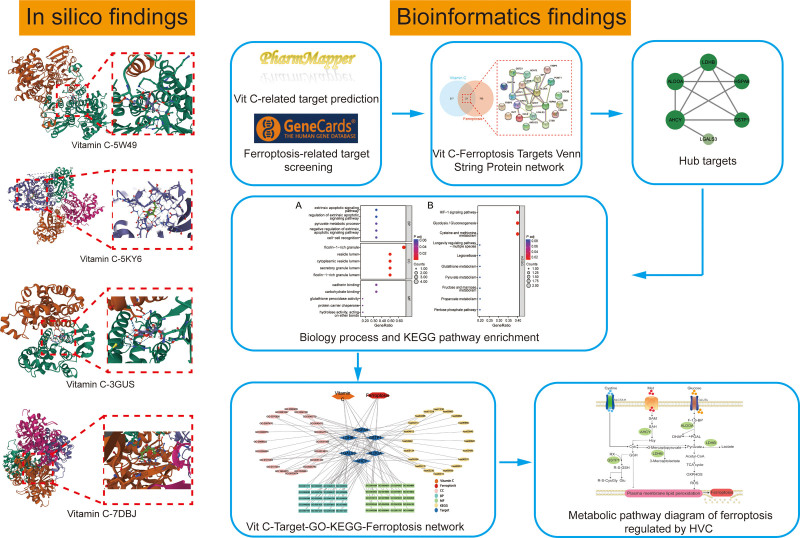
A schematic diagram of network pharmacology approach for the identification of core targets, PPI network, biological processes and key pathways in the regulation of ferroptosis by Vc. All known Vc- and ferroptosis-related targets were predicted from online databases. Then, potential targets for the regulation of ferroptosis by Vc were identified. After constructing the PPI network and identifying the core targets of Vc action on ferroptosis, GO term and KEGG pathway enrichment analyses were performed. In addition, maps of the Vc-Target-GO-KEGG-Ferroptosis network and the metabolic pathways of HVC-regulated ferroptosis were generated. Finally, the core targets were validated by molecular docking techniques. GO = gene ontology, HVC = high-dose vitamin C, KEGG = Kyoto Encyclopedia of Genes and Genomes, PPI = protein-protein interaction, Vc = vitamin C.

Network pharmacology integrates the disciplines of systems biology, bioinformatics, and network science to analyze the molecular links between drugs and their targets from the overall perspective of system level and biological network. It also reveals the systemic pharmacological mechanisms of drugs to guide the development of new drugs and clinical diagnosis and treatment, which is an emerging discipline in the systematic research of drugs in the era of artificial intelligence and big data. Network pharmacology is considered the “next generation of drug research.^[9]^ Our study integrates various database resources to investigate the regulatory mechanisms of Vc in ferroptosis from a holistic standpoint through multi-level analyses, encompassing target identification, construction of protein-protein interaction networks, and enrichment of signaling pathways. This approach provides a foundational framework for the clinical implementation of high-dose Vc antitumor therapy. In addition, the Vc targets identified using bioinformatics were further confirmed using molecular docking techniques. The workflow is illustrated in Figure 1.

## 2. Materials and methods

### 2.1. Vc-related target prediction

The 2D structure of Vc was saved as an SDF file in the PubChem database (https://pubchem.ncbi.nlm.nih.gov/) for target prediction. Vc-related targets were obtained from the PharmMapper database (http://www.lilab-ecust.cn/pharmmapper/).^[10]^ The obtained targets were also standardized using the UniProt database (https://www.uniprot.org/),^[11]^ with the species restricted to “Homo sapiens.”

### 2.2. Ferroptosis-related target screening

The ferroptosis-related targets were mined from the GeneCards database (https://www.genecards.org).^[12]^ A higher score for a target in the GeneCards database represents a closer association with a disease or physiopathological process.

### 2.3. Vc-regulated ferroptosis target PPI network construction and core target screening

To clarify the interactions between Vc-related targets and ferroptosis-related targets, the intersection of the 2 targets was determined, and a Venn diagram was drawn.^[13]^ The intersection targets were submitted to STRING11.5 database (https://string-db.org) to construct the protein-protein interaction (PPI) network,^[14]^ with the biological species set to “homo sapiens.” The raw data were saved as tsv files. The PPI network was imported into the Cytoscape software (v3.9.1)^[15]^ and the Analyze Network plugin was used to screen Vc-regulated ferroptosis core targets based on degrees of freedom. The bioinformatics algorithm employed the Degree Value, where the upper limit of the filtering range corresponded to the maximum degree of freedom inferred from the topology data, while the lower limit corresponded to the average degree of freedom.^[16,17]^

### 2.4. Functional and pathway enrichment analyses of core targets

Gene ontology (GO) and Kyoto Encyclopedia of Genes and Genomes (KEGG) enrichment analyses of core targets and visualization of the corresponding results were performed using R language packages, including org.Hs.e.Db, ClusterProfiler^[18]^ and ggplot2. GO data were collected using the org.Hs.e.Db package. The threshold for the corrected *P* values was set at .05. The results of GO and KEGG enrichment analyses are presented using bubble plots.

### 2.5. Network visualization and signaling pathway summary

The major GO functions, including biological processes, cellular components, and molecular functions, and the major KEGG pathways involved in the core targets were mapped in the same network diagram using Cytoscape software (v3.9.1).^[19]^ Download maps of core target-enriched signaling pathways from the KEGG database. Combined with literature mining, these signaling pathway maps were screened and streamlined to summarize the parts of these signaling pathways related to the core target regulation of ferroptosis and crosstalk between signaling pathways in the same pathway map.

### 2.6. Molecular docking determination

To assess the binding energy and interaction patterns between Vc and its targets, we used Autodock Vina (v1.2.2) (http://autodock.scripps.edu/),^[20]^ a computerized protein-ligand docking software. The molecular structure of Vc was obtained from the PubChem Compound Database. The 3D coordinates of proteins AHCY (PDB ID, 5W49; resolution, 2.40 Å), ALDOA (PDB ID, 5KY6; resolution, 1.94 Å), GSTP1 (PDB ID, 3GUS; resolution, 1.53 Å), and LDHB (PDB ID, 7DBJ; resolution, 1.55 Å) were downloaded from the PDB database (https://www.rcsb.org/). The scope of molecular docking can be categorized into whole proteins and small-molecule docking positions already in the protein PDB file, with the latter being the preferred approach. Therefore, when choosing a protein PDB file, in addition to considering the resolution, files with existing small-molecule docking are preferred. We first prepared the protein and ligand files by converting all protein and molecular files into PDBQT format, removing all water molecules, and adding polar hydrogen atoms. The grid boxes were centered to cover the structural domains of each protein and accommodate free molecular motion. Molecular docking studies were performed using AutoDock Vina (V1.2.2).

## 3. Results

### 3.1. Acquisition of Vc- and ferroptosis-related targets

**Figure 2.**
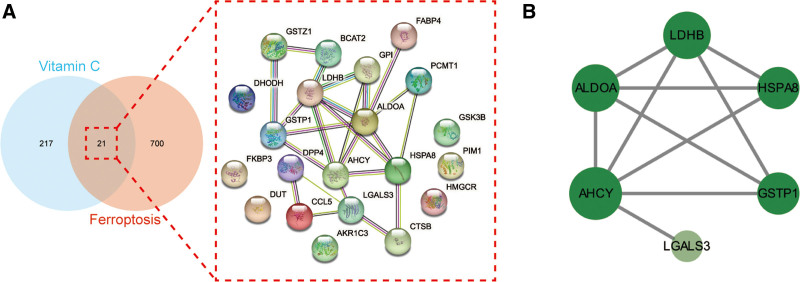
(A) As shown in the Venn diagram (left), all targets of Vc and ferroptosis were identified. In a PPI network (right), the merged targets of Vc and ferroptosis were interacted. (B) The core targets of Vc-regulated ferroptosis were interacted and visualized in a PPI network. PPI = protein-protein interaction, Vc = vitamin C.

A total of 238 Vc-related targets were obtained using PharmMapper database prediction, and 721 ferroptosis-related targets were obtained using the GeneCards database search (see Table S1, Supplemental Digital Content, which shows the targets of vitamin C and ferroptosis, http://links.lww.com/MD/M512). The Vc-related targets intersected with ferroptosis-related targets, and a total of 21 intersected targets were obtained (Fig. 2A).

### 3.2. PPI network construction and core target screening

An intersection target PPI network was constructed using the STRING database (Fig. 2A). The topological parameters of the PPI network were analyzed using the Analyze Network plug-in built into CytoScape software to obtain the core targets. The CytoScape network analysis showed that the maximum degree value of the target site was 7, and the mean degree value was 3.429. The range of degree values for screening core targets was set to 4 to 7. Six core targets were identified: fructose-bisphosphate aldolase A (ALDOA), adenosylhomocysteinase (AHCY), L-lactate dehydrogenase B chain (LDHB), heat shock cognate 71 kDa protein (HSPA8), galectin-3 (LGALS3), and glutathione S-transferase P (GSTP1) (Fig. 2B).

### 3.3. GO and KEGG enrichment analyses of core targets

**Figure 3.**
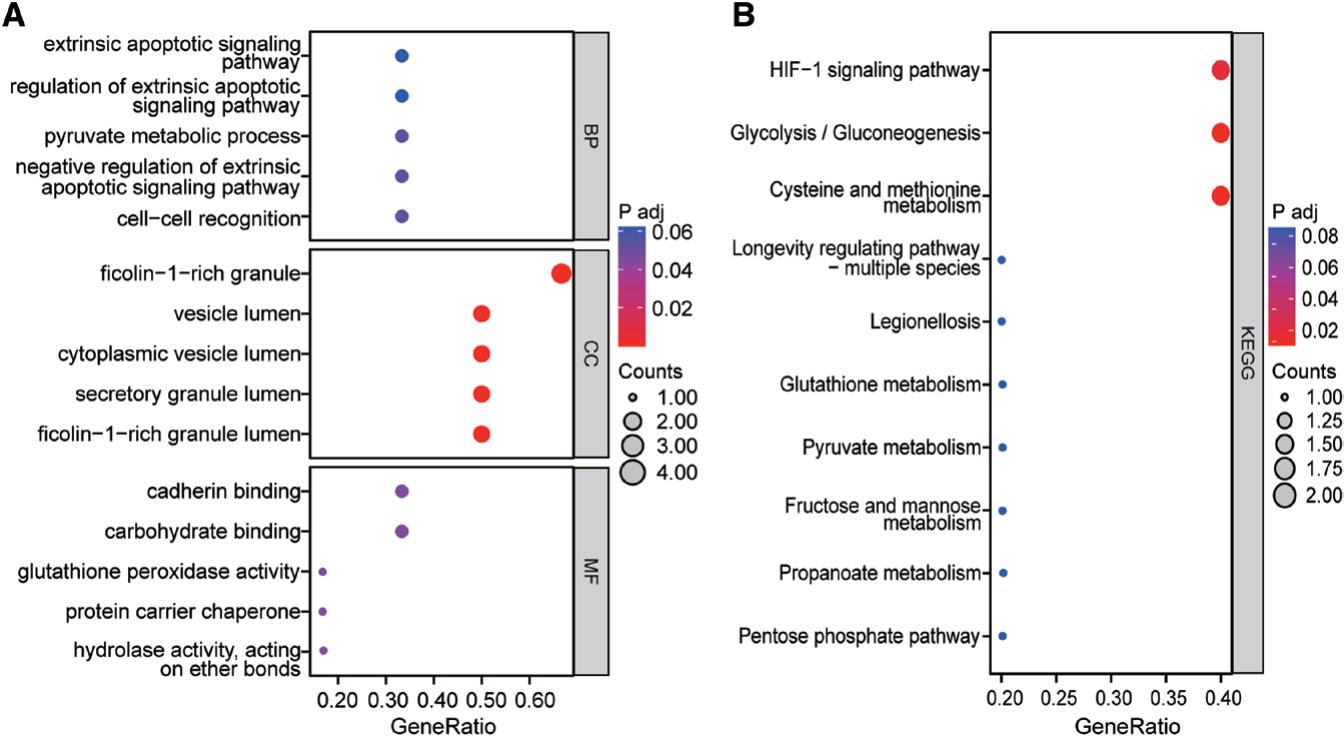
GO analysis (A) and KEGG analysis (B) of the core targets of Vc-regulated ferroptosis. All potential GO terms and KEGG pathways were detected and optimized, and the top-ranked molecular processes were highlighted in bubble diagrams. GO = gene ontology, KEGG = Kyoto Encyclopedia of Genes and Genomes, Vc = vitamin C.

GO function and KEGG pathway enrichment analyses were performed using 6 core targets, and the enrichment results were presented using bubble plots, yielding a total of 330 GO terms and 22 KEGG pathways (see Table S2, Supplemental Digital Content, which displays the enrichment results of GO and KEGG, http://links.lww.com/MD/M513). GO enrichment analysis suggests that the core targets are mainly involved in biological processes including extrinsic apoptotic signaling pathway, regulation of extrinsic apoptotic signaling pathway, pyruvate metabolic process, negative regulation of extrinsic apoptotic signaling pathway, cell-cell recognition, cell recognition, negative regulation of apoptotic signaling pathway, regulation of leukocyte proliferation, ATP metabolic process, T cell activation via T cell receptor contact with antigen bound to MHC molecule on antigen presenting cell, fructose 1,6-bisphosphate metabolic process, gas homeostasis, regulation of protein complex stability, negative regulation of acute inflammatory response, fructose metabolic process, leukocyte proliferation, immunological synapse formation, positive regulation by host of viral process, sulfur compound metabolic process, S-adenosylmethionine metabolic process, lactate metabolic process, negative regulation of JUN kinase activity and chaperone-mediated autophagy. Further, the main cellular components involved include ficolin-1-rich granule, vesicle lumen, cytoplasmic vesicle lumen, ficolin-1-rich granule lumen, secretory granule lumen, melanosome, pigment granule, tertiary granule, spliceosomal complex, ribbon synapse, Prp19 complex, clathrin-sculpted vesicle, postsynaptic cytosol, cytosolic region, sperm head, M band, mitochondrial inner membrane, A band, chaperone complex, luminal side of membrane, immunological synapse, terminal bouton, tertiary granule lumen and ficolin-1-rich granule membrane. In addition, the main molecular functions involved include carbohydrate binding, hydrolase activity, acting on ether bonds, cadherin binding, protein carrier chaperone, glutathione peroxidase activity, immunoglobulin binding, glutathione transferase activity, MHC class II protein complex binding, laminin binding, misfolded protein binding, MHC protein complex binding, chemoattractant activity, protein folding chaperone, protein phosphatase inhibitor activity, fatty acid binding, phosphatase inhibitor activity, carbon-carbon lyase activity, peroxidase activity, extracellular matrix binding, oxidoreductase activity, acting on peroxide as acceptor, transferase activity, transferring alkyl or aryl (other than methyl) groups and phosphatidylserine binding (Fig. 3A).

Moreover, KEGG enrichment analysis suggests that the core targets are mainly involved in pathways including Cysteine and methionine metabolism, Glycolysis/Gluconeogenesis, HIF-1 signaling pathway, Pentose phosphate pathway, Propanoate metabolism, Fructose and mannose metabolism, Pyruvate metabolism, Glutathione metabolism, Legionellosis, Longevity regulating pathway-multiple species, Chemical carcinogenesis-DNA adducts, Central carbon metabolism in cancer, Drug metabolism-cytochrome P450, Platinum drug resistance, Biosynthesis of amino acids, Metabolism of xenobiotics by cytochrome P450, Antigen processing and presentation, Drug metabolism-other enzymes, Prostate cancer, Glucagon signaling pathway, Toxoplasmosis and Carbon metabolism (Fig. 3B).

### 3.4. Interaction network visualization and signaling pathway summary map

**Figure 4.**
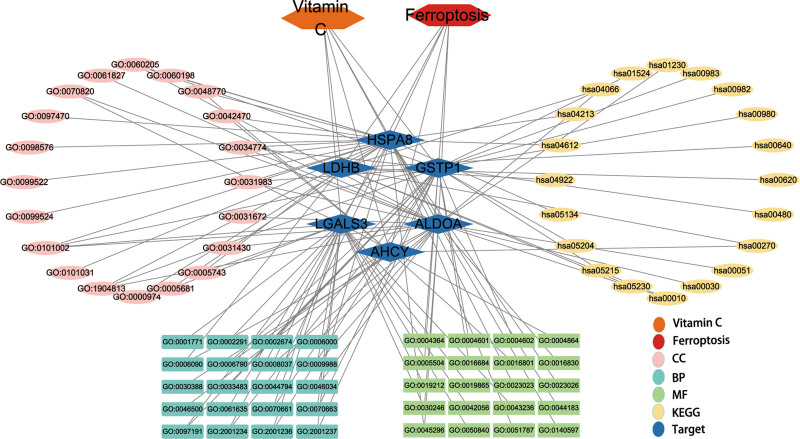
Interaction network based on the core targets of Vc-regulated ferroptosis. This diagram comprehensively reveals the interaction of targets, functions and pathways of the activities of Vc in regulating ferroptosis. Vc = vitamin C.

**Figure 5.**
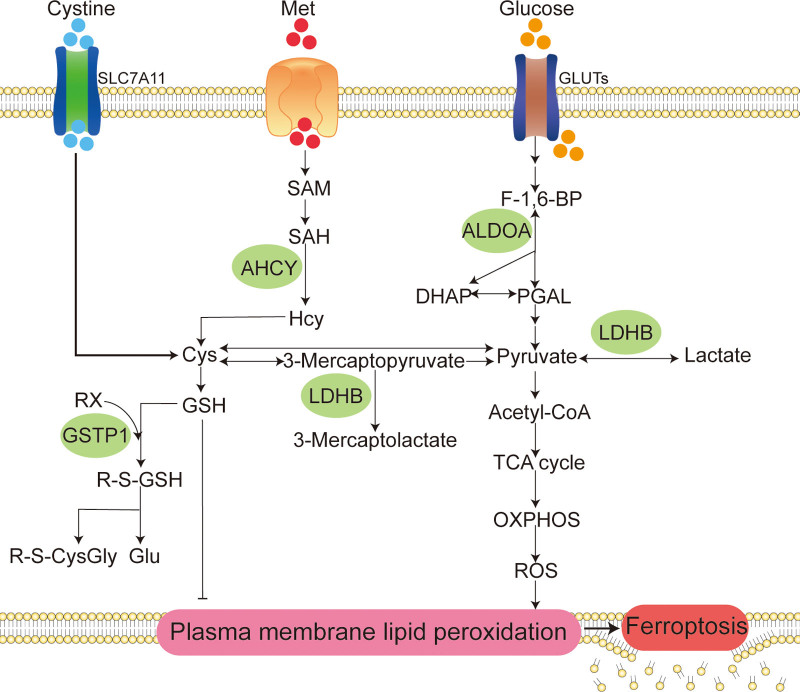
Metabolic pathway diagram of ferroptosis regulated by HVC. The potential mechanisms of HVC to regulate ferroptosis are the following three metabolic pathways: (A) targeting transsulfuration pathway to limit the supplemental synthesis of GSH; (B) targeting glycolysis to rewire glucose metabolism to the tricarboxylic acid cycle (TCA cycle) and oxidative phosphorylation (OXPHOS) to increase ROS production; and (C) targeting the reaction of GSH with organic halides to increase GSH catabolism utilization. In addition, HVC may exert antitumor effects by targeting ALDOA to limit the high utilization of glucose by tumor cells. Cys = cysteine, DHAP = dihydroxyacetone phosphate, F-1,6-BP = fructose-1,6-bisphosphate, Glu = glutamate, GLUTs = glucose transporters, GSH = reduced glutathione, Hcy = L-homocysteine, Met = methionine, PGAL = glyceraldehyde 3-phosphate, R-S-CysGly = R-S-cysteinylglycine, R-S-GSH = R-S-glutathione, RX = organic halide, SAH = S-adenosyl-L-homocysteine, SAM = S-adenosyl methionine, SLC7A11 = solute carrier family 7 member 11.

To demonstrate the bioinformatics findings, a core target-based interaction network was created and described in detail (Fig. 4). To visualize the signaling pathways of ferroptosis regulation by core targets, based on literature mining, the parts of KEGG signaling pathways enriched by core targets related to ferroptosis regulation by core targets and the parts related to the crosstalk between signaling pathways are summarized in Figure 5. As shown in the figure, core targets regulate ferroptosis mainly through metabolic pathways.

### 3.5. Molecular docking information

**Figure 6.**
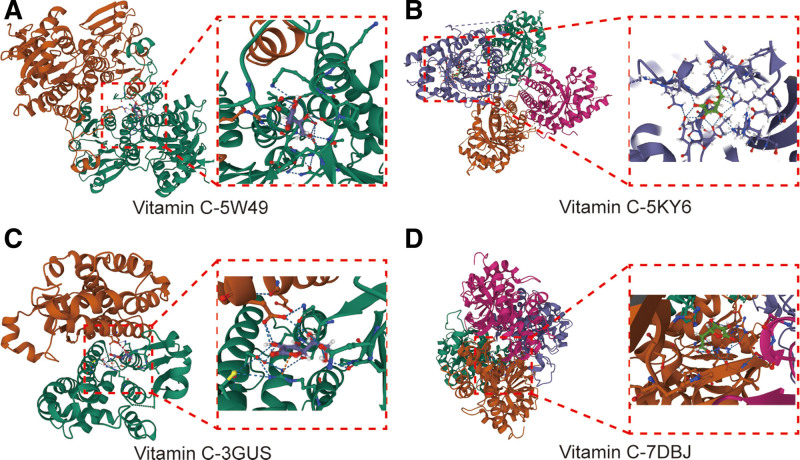
The molecular docking findings of Vc-regulated Ferroptosis. The core proteins of AHCY (PDB: 5W49), ALDOA (PDB: 5KY6), GSTP1 (PDB: 3GUS) and LDHB (PDB: 7DBJ) were identified to docking with Vc compound structurally. Vc = vitamin C.

To assess the affinity of Vc for its targets, we performed a molecular docking analysis. Binding poses and interactions of Vc with the four proteins were obtained using AutoDock Vina (v1.2.2), and binding energies for each interaction were generated. The results showed that Vc binds to its protein targets through visible hydrogen bonding and strong electrostatic interactions (Fig. 6). For Vc, four proteins, AHCY (PDB: 5W49), ALDOA (PDB: 5KY6), GSTP1 (PDB: 3GUS), and LDHB (PDB: 7DBJ), possessed good binding stability with binding energies of −5.44, −6.60, −4.96, and −5.41 kcal/mol, respectively.

## 4. Discussion

Numerous preclinical and clinical investigations have highlighted the efficacy of Vc in antitumor therapy.^[4]^ HVC can target multiple vulnerabilities that many cancer cells share.^[21]^ However, despite its potential, there is a pressing need for broader awareness and recognition of this highly promising nontoxic cancer treatment. In recent years, ferroptosis, a unique form of cell death, has garnered significant interest among tumor researchers. It is a type of cell death that can be induced by various therapeutic approaches, including radiation therapy,^[22]^ chemotherapy,^[23]^ and targeted therapy.^[24]^ Consequently, the induction of ferroptosis holds immense potential for tumor therapy. Notably, several studies have identified ferroptosis as a potential mechanism of antitumor therapy involving HVC.^[7,8]^ Therefore, the combination of HVC and ferroptosis induction presents a compelling avenue for innovative and effective antitumor treatments. Further exploration and research in this realm have the potential to unveil new opportunities for enhancing cancer treatments, thereby contributing to improved patient outcomes.

In this study, an in-depth exploration was conducted to elucidate the mechanisms underlying antitumor therapy of HVC based on Vc network targets. Through the construction of a Vc-regulated ferroptosis core target action network map, we discovered that HVC engages in multiple biological processes and signaling pathways by acting on various core targets. This intricate network constitutes a multipathway regulatory model for the modulation of ferroptosis. Among the top ten enriched pathways (Fig. 3B) identified by the core targets, two amino acid metabolic pathways and five carbohydrate metabolic pathways were prominent. This observation suggests that HVC primarily enhances ferroptosis by impacting metabolic pathways. Studies have shown that ferroptosis is intrinsically linked to multiple cellular metabolic pathways, including energy, amino acid, and lipid metabolism.^[25–28]^ These metabolic pathways play a direct role in determining the susceptibility of cells to lipid peroxidation and subsequent ferroptosis. The insights gained from this study shed light on the complex interplay among HVC, ferroptosis, and cellular metabolism, offering valuable perspectives into the mechanisms at play in the antitumor effects of HVC.

To further visualize the findings of the signaling pathway enrichment analysis, we combined literature mining to summarize and streamline core target-enriched signaling pathways. As shown in the summary figure (Fig. 5), four of the six core targets, specifically AHCY, LDHB, GSTP1, and ALDOA, regulate ferroptosis through metabolic pathways. We inferred three different mechanisms by which HVC promotes ferroptosis in cancer cells.

First, HVC may limit reduced glutathione (GSH) synthesis by targeting the transsulfuration pathway. AHCY is the only known enzyme that catalyzes the hydrolysis of S-adenosyl-L-homocysteine to L-homocysteine (Hcy) via the transsulfuration pathway.^[29]^ Hcy can also produce the precursor cysteine of GSH.^[30]^ GSH, an important reducing substance in the body, is involved in the most important defense mechanism against ferroptosis, the GPX4-GSH system.^[31]^ There are two main pathways for the intracellular production of cysteine: the cystine input pathway through solute carrier family 7 member 11^[32,33]^ and the transsulfuration pathway.^[34]^ Under conditions of insufficient cystine input, cells maintain GSH synthesis through Hcy production, thereby relieving ferroptosis stress. Cao et al found that inhibition of AHCY can synergize with inhibitors of solute carrier family 7 member 11, such as erastin, to promote ferroptosis.^[34]^ Therefore, we speculate that the combined application of HVC and type I ferroptosis inducers can greatly improve the induction of ferroptosis. Second, HVC may increase ROS production by targeting glycolysis to restore glucose metabolism to the TCA cycle and oxidative phosphorylation (OXPHOS). LDHB is a subunit of lactate dehydrogenase that catalyzes the biochemical conversion of pyruvate to lactate and 3-mercaptopyruvate to 3-mercaptolactate. Tumor cells show increased glycolytic dependence and exhibit elevated glucose uptake and fermentation of glucose to lactate to meet the high anabolic demands of cancer cell proliferation and maintain redox homeostasis.^[35,36]^ Meanwhile, mitochondrial OXPHOS activity in glycolytic tumor cells is significantly inhibited to alleviate ROS stress.^[35]^ However, glycolysis-induced inhibition of OXPHOS is not permanent and may reverse when glycolysis is inhibited, accompanied by increased sensitivity to ferroptosis inducers.^[37]^ HVC targeting LDHB may restore tumor cell glucose metabolism from glycolysis to the TCA cycle and OXPHOS, increasing ROS production to promote ferroptosis. Finally, HVC may increase the catabolic utilization of GSH by targeting the reaction between GSH and organic halides. GSTP1 is involved in binding GSH to a large number of exogenous and endogenous hydrophobic electrophiles.^[38]^ GSTP1 is involved in the enzymatic reaction of GSH with an organic halide to produce halide anions and S-substituted glutathione. S-substituted glutathione was further broken down to produce R-S-cysteinylglycine and glutamate. HVC may inhibit the most important defense pathway for ferroptosis: the GPX4-GSH pathway, by targeting GSTP1 and promoting the catabolic utilization of GSH.

At the end of the study, we validated the molecular targets of Vc regulation using molecular docking. Molecular docking is a computational chemistry method used to predict the binding mode and capacity between small molecules and target proteins. It is widely used in drug discovery, biochemical research, and bioinformatics. The goal of molecular docking is to determine the optimal binding mode of small molecules at the active site of a protein. By calculating and evaluating the interaction energies between small molecules with different conformations and proteins, their binding affinities and inhibitory activities can be predicted. Our results suggest that Vc binds to four proteins, AHCY, ALDOA, GSTP1, and LDHB, through a large number of visible hydrogen bonds, and yields low binding energies, suggesting good stability.

The execution of ferroptosis hinges on a pivotal process involving iron-catalyzed peroxidation of phospholipids rich in polyunsaturated fatty acids. This peroxidation event can result in perilous accumulation of lipid peroxides on cell membranes, eventually leading to membrane rupture when the ferroptosis drive system surpasses the buffering capacity of the defense system. Consequently, ferroptotic cell death occurred. Cellular amino acid and energy metabolic activities are involved in the regulation of key ferroptosis markers such as GSH and ROS, and thus play a potential regulatory role in ferroptosis. In tumor cells, adaptive metabolic responses act as a means of self-protection, enabling them to thwart ferroptosis. For instance, tumor cells may activate glycolysis, a process known as the Warburg effect, to impede ferroptosis. Both amino acid and energy metabolism have emerged as crucial targets for disrupting redox homeostasis and inducing ferroptosis. HVC, through distinct mechanisms, regulates both amino acid metabolism and energy metabolism, thereby facilitating lipid peroxidation while concurrently reducing cellular reducing capacity. This orchestrated interplay ultimately fosters ferroptosis.

In summary, while the study offers valuable insights into how HVC may promote ferroptosis in tumor cells, it is essential to address several limitations. Firstly, the lack of experimental validation for the proposed mechanism of action of HVC underscores the necessity for experimental studies to confirm the computational findings. Secondly, the study’s narrow focus on the mechanism of action of HVC in promoting ferroptosis in tumor cells overlooks other potential mechanisms or effects on tumor cells. It is hoped that more studies will be conducted in the future to complement the HVC anti-tumor mechanism. Thirdly, the selection of data from public databases may be biased, and it is hoped that more studies will be analyzed in the future using data from more sources to validate or correct our findings. Lastly, the study’s findings, based on computational analysis, may not fully capture the complex biological interactions in vivo, highlighting the importance of further research in animal models or humans.

## 5. Conclusions

This study identified three possible mechanisms by which HVC promotes ferroptosis in the context of tumor treatment based on a network pharmacology approach. We inferred that HVC promotes ferroptosis mainly through the regulation of amino acid and carbohydrate metabolism. In addition, this study found that the combination of HVC and type I ferroptosis inducers might have synergistic effects, which can be used as a basis for developing new therapeutic combination strategies. HVC may have a stronger ferroptosis-inducing effect on glycolysis-dependent tumor cells, and this understanding will facilitate further sorting of the population that could benefit from HVC treatment. Further studies are recommended to validate the results of the present study.

## Author contributions

**Conceptualization:** Jinxiu Qu, Shuai Lu.

**Data curation:** Jinxiu Qu, Bing Wang.

**Formal analysis:** Shuai Lu, Bing Wang, Zhenpeng Yang, Huazhen Tang.

**Funding acquisition:** Benqiang Rao.

**Investigation:** Jinxiu Qu, Bing Wang, Shiwan Wang, Huazhen Tang.

**Methodology:** Jinxiu Qu, Shuai Lu, Zhenpeng Yang, Huazhen Tang, Yuan Zhao, Xin Wang.

**Project administration:** Jinxiu Qu.

**Resources:** Yuan Zhao, Xiaozhu Liu.

**Software:** Jinxiu Qu, Bing Wang, Shiwan Wang, Jia He, Yuan Zhao, Xin Wang, Xiaozhu Liu.

**Supervision:** Jia He, Xiaozhu Liu, Benqiang Rao.

**Validation:** Shuai Lu, Shiwan Wang, Huazhen Tang, Jia He, Yuan Zhao, Benqiang Rao.

**Visualization:** Jinxiu Qu, Shiwan Wang, Zhenpeng Yang, Jia He, Xin Wang, Xiaozhu Liu.

**Writing – original draft:** Jinxiu Qu, Shuai Lu, Zhenpeng Yang.

**Writing – review & editing:** Yuan Zhao, Xin Wang, Xiaozhu Liu, Benqiang Rao.

## Supplementary Material
